# Predictive Ability of Neutrophil-Lymphocyte Ratio in Determining Tumor Staging in Colorectal Cancer

**DOI:** 10.7759/cureus.19025

**Published:** 2021-10-25

**Authors:** Chirag Pereira, Jiju Mohan, Shankar Gururaj, Prajwal Chandrashekhara

**Affiliations:** 1 General Surgery, Father Muller Medical College and Hospital, Mangalore, IND

**Keywords:** systemic inflammatory response syndrome, cancer metastasis, colorectal neoplasia, neutrophil to lymphocyte ratio (nlr), colorectal cancer

## Abstract

Introduction

Tumor staging plays an important role in determining treatment in colorectal cancer. In the recent past, the neutrophil-lymphocyte ratio (NLR) has been used as a predictive marker of inflammation for different types of clinical entities. Our study aims to determine if NLR can predict tumor staging in patients with colorectal cancer.

Materials and methods

We retrospectively analyzed all cases that underwent surgical treatment for colorectal cancer from 2014 to 2020. The NLR, tumor stage, and histology report for all patients were reviewed. Recommended cut-off values for NLR for tumor stage (T), lymph node stage (N), and metastatic stage (M) were determined using receiver operating characteristic (ROC) analysis.

Results

NLR was found to be significantly higher in patients with T3-T4 tumors as compared to T1-T2 tumors (mean: 5.8 vs. 2.6, respectively p < 0.001). The NLR values were higher in cases of N1-N2 groups as compared to N0 groups (mean: 5.7 vs. 3.5, p = 0.07). The NLR was also higher in M1 patients as compared to M0 patients (32.1 vs. 4.5, respectively, p = 0.24) but failed to show a statistical significance.

Conclusion

NLR is a useful predictor of colorectal cancer which can give us some information about the type of tumor we may encounter during surgery.

## Introduction

Tumour, node, metastasis (TNM) classification plays an important role in predicting the prognosis of various malignancies including colorectal cancer (CRC). There have been other markers such as carbohydrate antigen (CA19-9) and carcinoembryonic antigen (CEA) which have been utilized to assess tumor prognosis and evaluate chances of recurrence [1,2]. Recent studies have indicated that the body hosts immune response to tumor cells which can cause markers of inflammation to get elevated. Inflammatory markers like elevated C-reactive protein (CRP) have shown to be associated with a poor prognosis in cancers [3]. Neutrophil-lymphocyte ratio (NLR) is also an inflammatory status indicator that has been used in assessing prognosis in gastric, breast, and pancreatic cancers [4-6]. In this study, we investigated the predictive ability of preoperative NLR for tumor staging in CRC.

## Materials and methods

We retrospectively analyzed the digital records of all patients who were diagnosed with CRC and had undergone hemicolectomy, anterior resection, or abdominoperineal resection for CRC from January 2014 to December 2020 at Father Muller Medical College and Hospital, Mangalore, India. Exclusion criteria included those patients whose records were incomplete, received preoperative chemotherapy, history of recent blood transfusions, immunocompromised patients, patients with hematological disease or chronic inflammatory disease.

Based on the above criteria, among 270 patients who underwent colorectal surgery, 67 patients had to be excluded leaving behind 202 patients for our study. The NLR was calculated by dividing the percentage of neutrophils by lymphocytes from the peripheral WBC count. The patient’s age, sex, NLR, tumor localization, staging, and histological grade were recorded. Patients were staged based on American Joint Committee on Cancer (AJCC) TNM system.

Statistical analysis

**Figure 1:**
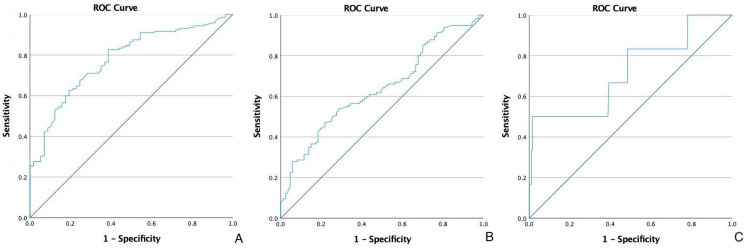
ROC curve analysis of the neutrophil-to-lymphocyte ratio in patients with colorectal cancer for T stage (A), N stage (B), and M stage (C). ROC: Receiver operating characteristic.

Data were analyzed using IBM SPSS for mac version 25. Data were shown as mean ± SD or median where applicable. The differences between groups were compared by using the student t-test and categorical data were analyzed by the Pearson chi-square test where appropriate. A p-value of less than 0.05 was considered statistically significant. Receiver operating characteristic (ROC) analysis was used to obtain cut-off values to discriminate between various groups. Based on ROC analysis, an NLR cut-off of 2.6 was used to distinguish the early T stage (T1-T2) from the advanced T stage (T3-T4). The area under the ROC curve was 0.779 (95% CI: 0.712-0.846, p < 0.001). An NLR cut-off of 3.3 was used to distinguish N0 from N1-N2 with the area under the ROC curve being 0.639 (95% CI: 0.563-0.715, p < 0.001). For distant metastasis, an NLR of 3.1 was used to distinguish M0 from M1. The area under the ROC curve was 0.720 (95% CI: 0.481-0.959, p = 0.067) (Figure 1).

## Results

A total of 202 patients were included in this study. The mean age was 56.2 ± 13.7 years, with age ranging from 25 to 87 years. There were 109 (48.2%) males and 117 (51.8%) females in the study. The most common site for the tumor was in the rectum (39.2%) while the least common site was in the anal canal (2.9%). There were synchronous localization of tumors in two (0.9%) patients (Table 1). In both cases, the tumors were situated in the ascending colon and the transverse colon.

**Table 1:** Tumor localization of patients with colorectal cancer.

Tumor localization	n	%
Anal canal	6	2.9
Rectum	79	39.2
Left colon	69	34.2
Transverse colon	11	5.4
Right colon	35	17.4
Synchronous tumors	2	0.9
Total	202	100

In relation to tumor depth, we found a significantly higher NLR value for T3-T4 tumors as compared to T1-T2 tumors (mean: 5.8 vs. 2.6, p < 0.001). The diagnostic accuracy of NLR at this cut-off was 75.2%. When evaluating NLR based on lymph nodal involvement, the NLR was higher in N1-N2 groups as compared to N0 group (mean: 5.7 vs. 3.5, p = 0.07) but it failed to show any statistical significance. A NLR cut-off of 3.1 was used to distinguish metastatic from non-metastatic disease. The mean NLR was 32.1 for metastatic disease which was higher than non-metastatic disease i.e., 4.5. However, this failed to show any statistical significance (Table 2).

**Table 2:** Comparing NLR between patient groups with different cancer stages Sen: Sensitivity; Spec: Specificity; PPV: Positive predictive value; NPV: Negative predictive value.

Colorectal Cancer		Mean	SD	Median	p-value	Cut-off value	Sen	Spec	PPV	NPV	Accuracy
Tumor depth	T1-T2	2.6	1.8	2.3	P < 0.001	2.6	82.7%	56.1%	82.7%	56.1%	75.2
	T3-T4	5.8	13.1	4.8							
Lymph node invasion	N0	3.5	4.9	3.3	P = 0.07	3.3	64.3%	48.2%	62.1%	50.6%	57.4
	N1-N2	5.7	14.2	4.8							
Distant organ metastasis	M0	4.5	9.7	3.8	P = 0.24	3.1	83.3%	40.3%	4.1%	98.7%	41.5
	M1	14.2	32.1	24.4							

## Discussion

CRC is the third most common cause of cancer-related deaths worldwide, with incidence rates higher in the older population [7]. This disease is more commonly diagnosed in men as compared to women [7] but we had more number of women in our study as compared to men. The sigmoid colon is the most common site of cancer in the colon while the rectum is the most common site of cancer in the lower gastrointestinal tract [8]. The most common site of cancer in our study was the rectum (39.2%) followed by the left colon (34.2%). Özgehan G et al. [9] in their study had the majority of tumors arising from the rectum (50.5%) followed by the sigmoid colon (19.4%).

The exact mechanism of elevated NLR and association with various cancers is not clearly understood. The immune system of the body plays an important role in determining the outcome of cancer [10,11]. Neutrophils promote tumor cellular proliferation, invasion as well as vascularization by means of releasing certain factors like chemokines. Hence, an elevated neutrophil count promotes tumor growth and metastasis. Lymphocytes, on the other hand, suppress tumor growth. Lymphocytes are cytotoxic in activity and kill tumor cells as well as produce cytokines that inhibit tumor proliferation and metastasis [12]. In summary, NLR is a reflection of the balance between anticancer immune status and pro-tumor inflammatory status.

In our study, we used NLR at a cut-off of 2.6 to distinguish the early T stage from the advanced T stage with the mean NLR being 2.6 and 5.8, respectively. Özgehan G et al. [9] used a similar cut-off of 2.3 to distinguish early from advanced T stage CRC but the mean NLR was higher for both early and advanced T stage (4.4 and 5.2, respectively). We used a cut-off of 3.3 and 3.1 for N stage and M stage, respectively. Although, the mean NLR was elevated in both cases of lymph nodal spread and tumor metastasis, it failed to show a statistical significance. Özgehan G et al. [9] used a cut-off of 3.2 and 3.4 for N and M stage, respectively and showed that NLR was statistically higher in both cases. They had more number of patients with metastatic disease as compared to study and this may be the reason for statistical significance.

He W et al. [13] studied NLR in metastatic colorectal cancer and found NLR to be significantly elevated. Tang H et al. [14] carried out a systemic review to evaluate NLR's relationship to colorectal liver metastasis. They found NLR to be significantly elevated and also found that an elevated pre-treatment NLR is associated with poor long-term survival. There have been numerous other studies that have utilized NLR to assess prognostic outcome in colorectal cancer. Jeon BH et al. [15] used NLR to assess predictive outcomes in patients with rectal cancer who underwent chemotherapy followed by surgery and found that preoperative elevated NLR was independently associated with decreased five-year disease-free survival. Mazaki J et al. [16] and Dell'Aquila E et al. [17] showed that an elevated NLR is an important prognostic marker in advanced colorectal disease. Due to lack of adequate patient follow-up, we were unable to assess the prognostic relationship between NLR and colorectal cancer in our patients.

## Conclusions

NLR is a marker of systemic inflammation and is a valuable predictive indicator of tumor invasion in CRC. An elevated NLR is highly suggestive of local tumor infiltration. NLR has also been shown to be elevated in lymph node spread and distant metastasis. There are numerous investigations available to assess tumor depth but the majority of which are expensive. NLR remains an inexpensive investigation that is easily available to the surgeon's hand. It gives an idea to the surgeon about what they might expect before opening up the abdomen.
